# A Receptor-based Switch that Regulates Anthrax Toxin Pore Formation

**DOI:** 10.1371/journal.ppat.1002354

**Published:** 2011-12-08

**Authors:** Rosemarie M. Pilpa, Monika Bayrhuber, John M. Marlett, Roland Riek, John A. T. Young

**Affiliations:** 1 Nomis Center for Immunobiology and Microbial Pathogenesis, The Salk Institute for Biological Studies, La Jolla, California, United States of America; 2 Structural Biology Laboratory, The Salk Institute for Biological Studies, La Jolla, California, United States of America; 3 Laboratory of Physical Chemistry, ETH Zürich, Zürich, Switzerland; University of Illinois, United States of America

## Abstract

Cellular receptors can act as molecular switches, regulating the sensitivity of microbial proteins to conformational changes that promote cellular entry. The activities of these receptor-based switches are only partially understood. In this paper, we sought to understand the mechanism that underlies the activity of the ANTXR2 anthrax toxin receptor-based switch that binds to domains 2 and 4 of the protective antigen (PA) toxin subunit. Receptor-binding restricts structural changes within the heptameric PA prepore that are required for pore conversion to an acidic endosomal compartment. The transfer cross-saturation (TCS) NMR approach was used to monitor changes in the heptameric PA-receptor contacts at different steps during prepore-to-pore conversion. These studies demonstrated that receptor contact with PA domain 2 is weakened prior to pore conversion, defining a novel intermediate in this pathway. Importantly, ANTXR2 remained bound to PA domain 4 following pore conversion, suggesting that the bound receptor might influence the structure and/or function of the newly formed pore. These studies provide new insights into the function of a receptor-based molecular switch that controls anthrax toxin entry into cells.

## Author Summary

The bacterium that causes anthrax produces a toxin called anthrax toxin that is largely responsible for causing disease symptoms. The first step in anthrax intoxication involves binding of the toxin to a specific protein, called a receptor, on the cell surface. Receptor-binding acts like a switch to prevent the toxin from forming a pore in a cell membrane until the toxin-receptor complex is taken up into cells and delivered to a specific location (called an endosome) where it is exposed to an “acid bath”. This acidic environment promotes structural changes in the toxin leading to pore formation in the endosomal membrane. In this report, we have studied how the receptor regulates pore formation by following the associated changes in toxin-receptor contacts. These studies have defined a new toxin-receptor intermediate in the pathway leading to pore conversion and demonstrate that the receptor remains bound after pore conversion. Our results provide important new insights into how the receptor regulates anthrax toxin pore formation, information that could be useful for designing new therapeutic strategies to treat this disease.

## Introduction

Cellular receptors can act as molecular switches that initiate conformational changes in microbial proteins required for cellular entry. Examples of such switches include an anthrax toxin receptor (described in detail below) as well as those for a number of viruses including HIV-1 and other retroviruses [1], [2], [3], measles virus [4], and herpesviruses [5]. The mechanisms by which these receptor-based switches function to promote cellular entry are only partially understood. In this report we set out to define the mechanism by which a receptor-based switch regulates anthrax toxin prepore-to-pore conversion.

Anthrax toxin, the key virulence factor secreted by *Bacillus anthracis,* is a bacterial AB toxin composed of three independent, plasmid-encoded polypeptide chains: the receptor-binding (B) moiety, protective antigen (PA), and two different enzymatic (A) moieties, lethal factor (LF) and edema factor (EF) [6], [7], [8]. The first step in cellular intoxication involves binding of an 83 kD form of PA (PA_83_) to specific cell surface receptors. Although several PA receptors have been defined [9], [10], [11], anthrax toxin receptor type 2 (ANTXR2) (also known as capillary morphogenesis protein 2; CMG2), is the most physiologically relevant receptor [12], [13], [14]. ANTXR2 is a type 1 transmembrane protein and its extracellular von Willebrand factor type A (VWA) domain is the site of PA-binding [15], [16]. Following receptor binding, PA_83_ is cleaved to a 63kD form (PA_63_) that spontaneously oligomerizes to form either a heptameric, or an octameric, PA_63_ prepore structure [17], [18]. Oligomeric PA_63_-receptor complexes are then taken into cells primarily by a clathrin-dependent endocytic mechanism and delivered to an acidic endosomal compartment where low pH triggers formation of a PA_63_ pore within an endosomal membrane [19], [20]. LF and EF are then translocated through the pore and delivered to the cytosol where they promote intoxication [21].

X-ray structural analysis of monomeric and heptameric PA-ANTXR2 VWA-domain complexes revealed that the receptor acts as a molecular switch or clamp that inhibits prepore-to-pore conversion at neutral pH [15], [16]. Specifically, the receptor VWA-domain interacts with the base regions of PA domains 2 and 4, thereby sterically hindering the movement of the PA 2β3-2β4 loop region necessary for pore formation [15], [16]. Those findings led to a model in which release of the receptor contact with PA domain 2 at an acidic endosomal pH is necessary to permit the conformational changes required for PA pore formation [15], [16]. Consistent with this idea, the pH threshold of the receptor-regulated toxin pore formation can be dictated by specific amino acid residues located at the PA domain 2-binding region of the ANTXR2 VWA-domain [22].

Presently, it is not clear if PA domain 2-receptor contacts are released at a step that occurs prior to, or is coincident with, prepore-to-pore conversion. Furthermore, it is not clear if the receptor remains attached following pore conversion and, if so, how it remains attached. Evidence supporting dissociation has come from co-immunoprecipitation experiments [23] and from previous NMR studies [24], [25]. On the other hand, evidence in favor of receptor attachment has come from other co-immunoprecipitation studies [19], [26], from NMR binding studies performed with a fragment (Domain 4) of PA [27], and from the finding that the presence of a receptor seems to influence voltage-dependent inactivation and small molecule inhibition properties of the newly formed pore [28]. Based upon structural considerations, it has also been argued that the receptor may remain bound to serve as a structural support for the pore [16], [29].

To clarify these issues, we have employed NMR techniques to monitor changes in the PA_63_ heptamer-ANTXR2 VWA domain contacts as a function of pH. Initially we attempted to examine the interaction between the ANTXR2 VWA domain and PA_63_ using chemical shift perturbation (CSP) by titrating in substoichiometric amounts of unlabeled PA_63_ into a ^1^H-^15^N labeled ANTXR2 sample. Based on results with other systems [30], we anticipated that titrating in PA_63_ might allow us to monitor chemical shift changes as a function of receptor binding and/or cause selective broadening of specific peaks associated with residues at the PA binding interface. If so, this would allow us to monitor specific receptor residues bound to PA_63_ under different pH conditions. Additionally, if shift perturbation of crosspeaks were detected using saturating conditions of the binding partner (PA_63_), this would help to approximate the fractional population of bound species versus the free species at equilibrium [30]. However, when low stoichiometric concentrations of PA_63_ were titrated into the ANTXR2-VWA domain sample, extensive line broadening and the disappearance of cross-peaks in the ^1^H-^15^N TROSY-HSQC was observed at a ratio of 1∶0.25 ANTXR2-VWA domain to PA_63_. This was likely due to the large size of the PA complex, indicating a larger effective correlation time (τ_c_), restricted local motion, and complete binding at the concentrations used. Therefore, we hypothesized that the method of transferred cross-saturation (TCS) may be well suited to investigate these interactions since this approach has previously been used to identify contact residues of protein ligands in large protein complexes [31].

To investigate the function of the ANTXR2-based switch, TCS was employed to monitor changes that occur in PA_63_ heptamer-ANTXR2 VWA-domain contacts as a function of pH. In this approach, an unlabeled protein is added at substoichiometric amounts to a deuterated, ^15^N-labeled protein, in this case, PA_63_ and the ANTXR2 VWA-domain, respectively. The aliphatic proton resonances of the unlabeled protein are then saturated with a brief radiofrequency pulse and this saturation is transferred selectively to contact residues of the ^2^H, ^15^N-labeled protein by spin diffusion. Consequently, the intensity of amide cross-peaks representing labeled residues that lie at the protein-protein interaction surface are selectively reduced by cross-relaxation [32], [33]. Here we have used this technique to obtain evidence for a new toxin-receptor intermediate in the pathway leading to pore formation and show that the receptor remains attached to PA domain 4 following low pH-dependent conversion. Additionally, chemical shift perturbations associated with receptor residues located near the PA domain 4 binding region revealed moderate conformational changes that occur during the attachment and detachment of PA from the receptor.

## Results

### Generating the deuterated, ^15^N-labeled ANTXR2 VWA-domain

**Figure 1:**
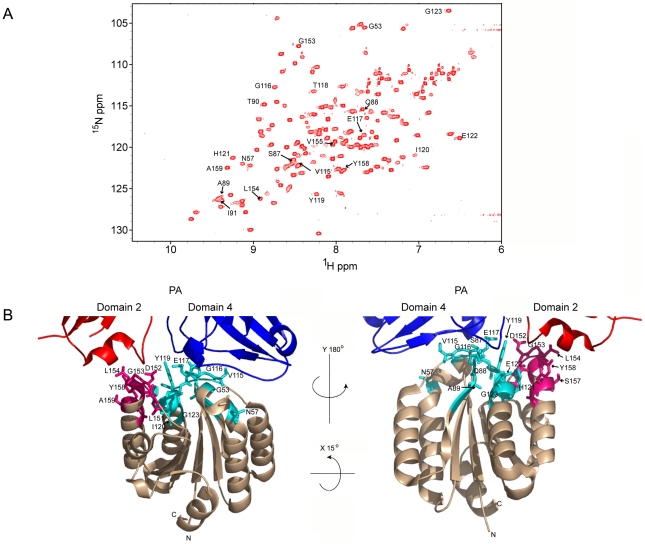
A) ^1^H-^15^N TROSY-HSQC of the ANTXR2 VWA domain. Cross-peaks with labeled assignments represent receptor residues at the PA domain 2 and domain 4 interaction surfaces. **B)** The crystal structure of the interface between monomeric PA_83_ bound to ANTXR2 (PDB 1T6B; Protein Data Bank), showing the base regions of domain 2 and 4 of PA. Representative PA contact residues of ANTXR2 are indicated: domain 2 contact sites are in hot pink and domain 4 contacts are in cyan. All images were generated using PymolX11 (DeLano Scientific, San Carlos, CA).

The deuterated, ^15^N-labeled ANTXR2 VWA-domain was produced as a GST-fusion protein from bacterial cells. In order to limit spin diffusion in the ^15^N-labeled protein, it was extensively deuterated by growing the cells in 100% D_2_O minimal media using ^2^H-glucose as the sole carbon source [32]. The labeled VWA-domain was purified to homogeneity as described under Materials and Methods and was unfolded to protonate the residues within the protein core and refolded to increase the number of cross-peaks in the [^15^N,^1^H] TROSY-HSQC spectrum. The integrity of the refolded protein was confirmed by circular dichroism (CD) analysis performed at either pH 8.0, 6.0, or 5.0 and in each case the protein displayed alpha-helical properties (Supplementary Figure S1). The refolded protein also functioned as an efficient receptor decoy in a toxin neutralization assay (Supplementary Figure S2). Moreover, Transverse Relaxation Optimized Spectroscopy-Heteronuclear Single-Quantum Coherence (TROSY-HSQC) spectrum analysis indicated that the protein was correctly refolded when compared to a control [^15^N, ^1^H] TROSY-HSQC spectrum of the ANTXR2 VWA domain that had not been previously denatured (Figure 1A). Assignments for the backbone resonances of the ANTXR2 VWA-domain were obtained using data from the following experiments: [^1^H-^15^N] TROSY-HSQC, 3D TROSY-HNCO, 3D TROSY-HN(CA)CO, 3D TROSY-HNCACB, 3D TROSY-HNCA, and a 3D ^15^N-edited NOESY-HSQC. NMR data were processed using NMRPipe and analyzed using Sparky and CARA software packages [34], [35], [36]. A representative example of this data analysis is shown in Supplementary Figure S3. Using this approach 87% of the backbone residues of the ANTXR2 VWA-domain, including the PA contact residues, were assigned (Figure 1A and B).

### ANTXR2 residues that contact PA domains 2 and 4 are saturated by transferred cross saturation (TCS)

**Figure 2:**
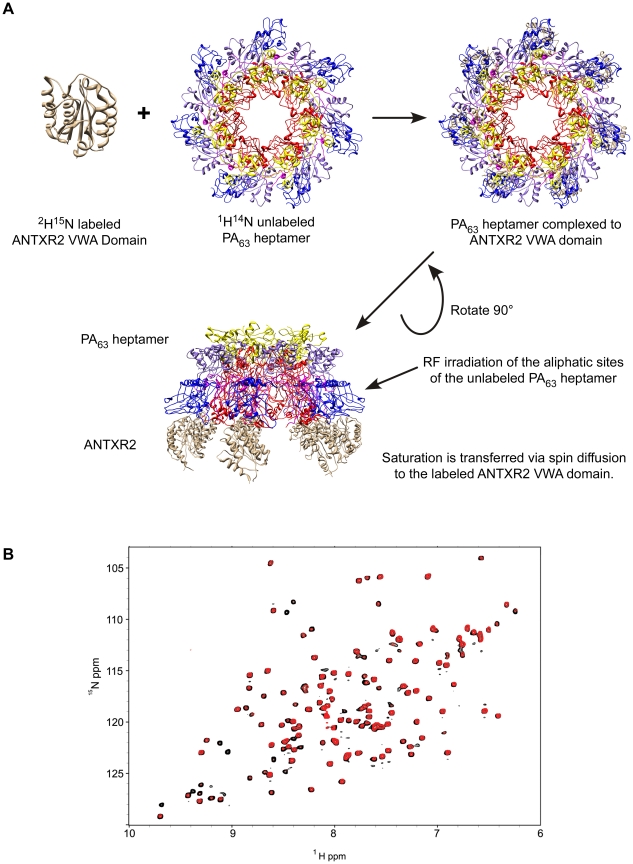
The principle of Transferred Cross Saturation (TCS) applied to the (PA_63_)_7_-ANTXR2 complex. **A**) Schematic of TCS between ANTXR2 VWA domain and the PA_63_ pore. ^2^H^15^N-labeled ANTXR2 VWA-domain was mixed at a ratio of (10∶1) with PA_63_ heptamer. Radiofrequency pulses were applied to the sample, in order to saturate the aliphatic protons of the PA_63_ heptamer. Saturation is then transferred to the contact residues of the labeled ANTXR2 VWA-domain, reducing the intensity of the corresponding cross peaks in the spectrum. **B**) Saturated and unsaturated spectra of the **(PA_63_)_7_**-ANTXR2 complex at pH 8.0. The [^1^H,^15^N] TROSY-HSQC spectra of the ^2^H-^15^N labeled ANTXR2-VWA domain complexed with the PA_63_ heptameric prepore are shown overlayed under saturating or non-saturating (black) conditions.

**Figure 3:**
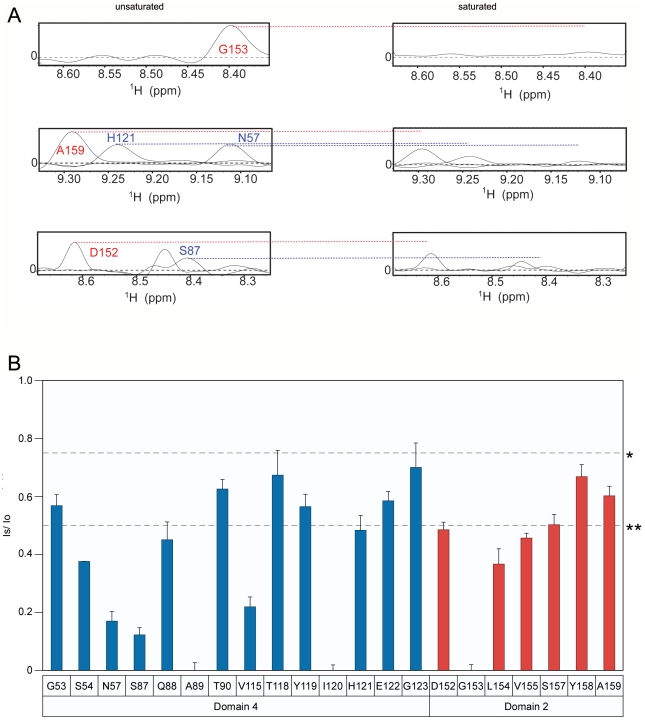
The receptor contact residues with PA domain 2 and 4 that are saturated at pH 8.0. **A**) A subset of the 1D slices of the [^15^N,^1^H] TROSY-HSQC spectra obtained at pH 8.0 highlighting several cross-peaks without saturation at pH 8 (left panels) or with saturation at pH 8 (right panels). Cross-peaks representing PA domain 2 and 4 contact residues are indicated with red and blue labels, respectively. **B**) A plot of the intensity ratio (I_s_/I_o_) of the transferred cross saturation of (PA_63_)_7_ and the interacting residues on the ANTXR2 VWA domain. Significant cross saturation (I_s_/I_o_≤0.75) is indicated with a single asterisk, and highly significant (I_s_/I_o_≤0.5) is indicated with a double asterisk. The errors were calculated by propagating the base-plane noise, which was derived from the signal-to-noise ratios of both control and the saturated spectra and this value was averaged from two duplicate experiments.

The principle of the TCS approach used to monitor PA_63_-ANTXR2 VWA-domain interactions is outlined in Figure 2A. In order to observe saturation transfer, the concentration of the binding partner must be kept sufficiently low to effectively allow for fast exchange so that amide cross-peaks are not broadened following its addition. The efficiency of TCS depends on the sample conditions as well as the binding constants between the receptor and PA. According to Shimada et al, TCS is applicable for a system where a large p_B_, or fraction of bound ligands is preferred for high saturation efficiency, if k_off_ >0.1 s^−1^, or if k_off_ ≥10 s^−1^, a p_B_ ≥0.1 is preferred [37]. Therefore, for the TCS experiments, the concentrations of the two protein partners were optimized by performing titration experiments at pH 8.0, and a (10∶1) molar ratio of the ANTXR2 VWA-domain to PA_63_ was chosen for all cross saturation experiments, because at this concentration there were no signs of peak broadening. Three separate sets of interleaved experiments were subsequently performed on the ANTXR2 VWA: (PA_63_)_7_ complex in buffers of pH 8.0, 6.0, and 5.0. Saturation transfer was achieved by applying a selective radiofrequency pulse at 0.8 ppm, prior to the [^15^N,^1^H] TROSY-HSQC. A pH of 8.0 was chosen for the initial analysis because it closely approximated those used previously for X-ray structural analysis of PA-receptor complexes. i.e. pH 7.5 [16] and pH 8.25, [15]. These studies revealed that the majority of the labeled residues in the ANTXR2 VWA-domain were not saturated by a radiofrequency pulse, i.e. those with similar signal intensities under conditions of no saturation (black peaks) or saturation (red peaks) (Figure 2B). However, a subset of the amide cross-peaks were saturated (black-only peaks) in the overlayed spectra (Figure 2B) and a number of those cross-peaks corresponded to contact residues with PA domains 2 or 4. For simplicity the saturation data was represented as 1D cross sections of the corresponding cross-peaks in the HSQC spectra (Figure 3A). The degree of saturation of each residue was calculated by dividing the observed peak intensity of the saturated spectrum (I_s_) by the observed peak intensity of the control spectrum (I_o_) (unsaturated). In these studies an (I_s_/I_o_) value of <0.75 is considered significant and one of <0.5 highly significant, as in [37]. Based upon these criteria all of the PA domain 2 and 4 contact residues that could be unambiguously assigned were saturated under these conditions (Figure 3B). Taken together, this study verified that the TCS method can be used to specifically monitor contacts between the ANTXR2 VWA-domain and PA domains 2 and 4 in the heptameric toxin-receptor complex.

### Identification of a new toxin-receptor complex intermediate at pH 6.0

To characterize the changes in PA-receptor contacts that occur after incubating the complex under mildly acidic conditions, the TCS experiment was repeated at pH 6.0. That condition is approximately 1.0 pH unit above that needed to trigger toxin prepore to pore conversion when the PA heptamer is bound to the ANTXR2 VWA-domain [23], [28] (Supplementary Figure S4). However, not all of the PA-contact residues that were observed at pH 8 were visible in the saturated and unsaturated spectra obtained at pH 6. This finding is probably due to a structural change upon loss of contact with the PA yielding to an increased H/D exchange or/and slow conformational exchange dynamics.

**Figure 4:**
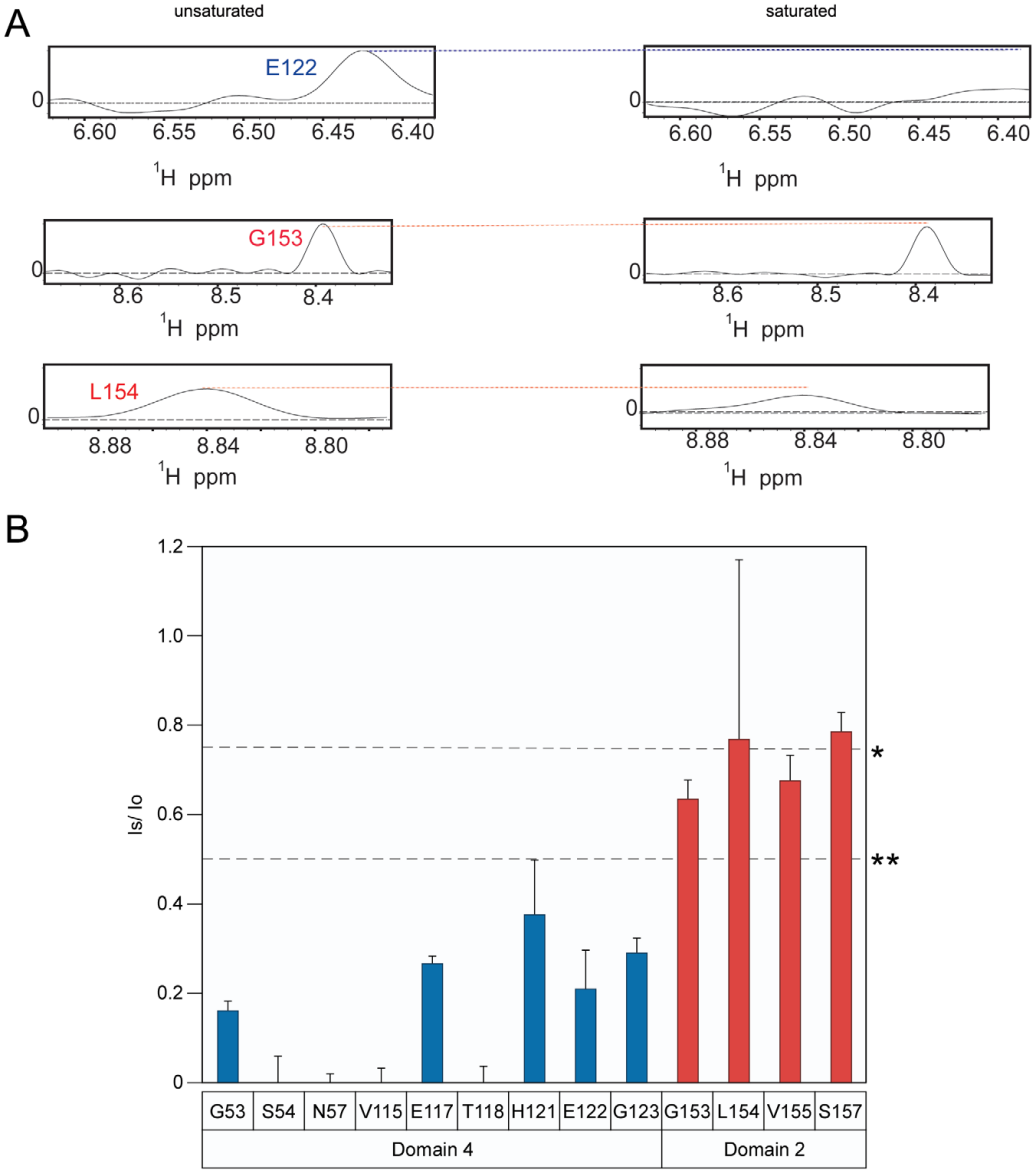
The receptor contact with PA domain 2 is weakened at pH 6.0. **A**) A subset of the 1D slices of the [^15^N,^1^H] TROSY-HSQC spectra obtained at pH 6.0 highlighting several cross-peaks without saturation at pH 6 (left panels) or with saturation at pH 6 (right panels). Cross-peaks representing PA domain 2 and 4 contact residues are indicated with red and blue labels, respectively. **B**) A plot of the intensity ratio (I_s_/I_o_) from the transferred cross saturation of (PA_63_)_7_ heptamer to interacting residues on the ANTXR2 VWA-domain. Significant cross saturation (I_s_/I_o_≤0.75) is indicated with a single asterisk, and highly significant (I_s_/I_o_≤0.5) is indicated with a double asterisk. The errors were calculated by propagating the base-plane noise, which was derived from the signal-to-noise ratios of both control and the saturated spectra and this value was averaged from two duplicate experiments.

Analysis of the data clearly showed that the PA domain 4 contact residues that were resolved remained strongly saturated at pH 6 (residues G53, S54, N57, V115, E117, T118, H121, E122, and G123; (Figure 4A and 4B). By striking contrast, PA domain 2 contact residues were much less saturated at pH 6.0 (Figure 4B). Taken together, these data are consistent with a model in which the receptor remains bound to PA domain 4 but its interactions with PA domain 2 are significantly weakened or are lost prior to prepore-to-pore conversion.

### The ANTXR2 VWA-domain remains attached to PA domain 4 following prepore-to-pore conversion

**Figure 5:**
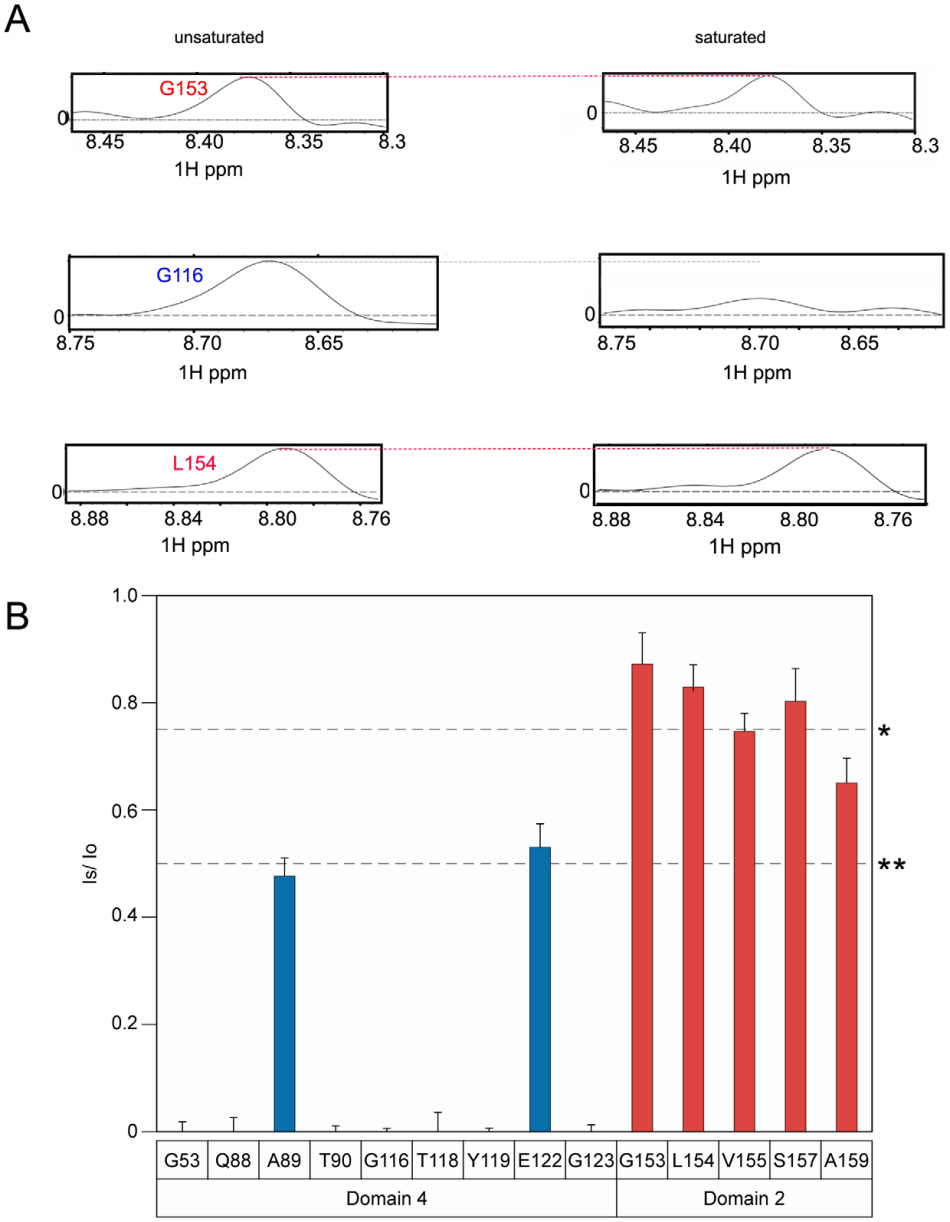
Receptor remains bound to PA domain 4 at pH 5.1. **A**) A subset of the 1D slices of the [^15^N,^1^H] TROSY-HSQC spectra highlighting several cross-peaks without saturation at pH 5.1 (left panels) or with saturation at pH 5.1 (right panels). Cross-peaks representing PA domain 2 and 4 contact residues are indicated with red and blue labels, respectively. **B**) A plot of the intensity ratio (I_s_/I_o_) from the transferred cross saturation of (PA_63_)_7_ to interacting residues on the ANTXR2 VWA domain. Significant cross saturation (I_s_/I_o_≤0.75) is indicated with a single asterisk, and highly significant (I_s_/I_o_≤0.5) is indicated with a double asterisk. For all graphs the errors were calculated by propagating the base-plane noise, which was derived from the signal-to-noise ratios of both interleaved experiments. The data was taken from two separate experiments performed at pH 5.1 and pH 5.15 and the average was derived from these experiments.

When bound to ANTXR2, the PA_63_ prepore is triggered to form a pore species at pH values that are less than or equal to pH 5.2 [23], [28] (Supplementary Figure S4). Therefore, to determine if the receptor remains attached to PA following pore formation, the TCS experiment was performed under both saturating and non-saturating conditions at pH 5.1. Consistent with the results obtained at pH 6.0, the receptor residues that contact PA domain 2 were not saturated at pH 5.1, with the possible exception of residue A159 (Figure 5A and 5B). More importantly however, virtually all of the domain 4 contact residues that could be resolved at this pH value were saturated at pH 5.1 (Figure 5B). These data are consistent with a model in which the receptor contacts with PA domain 2 are lost during anthrax toxin prepore to pore conversion but the receptor remains bound to PA domain 4.

**Figure 6:**
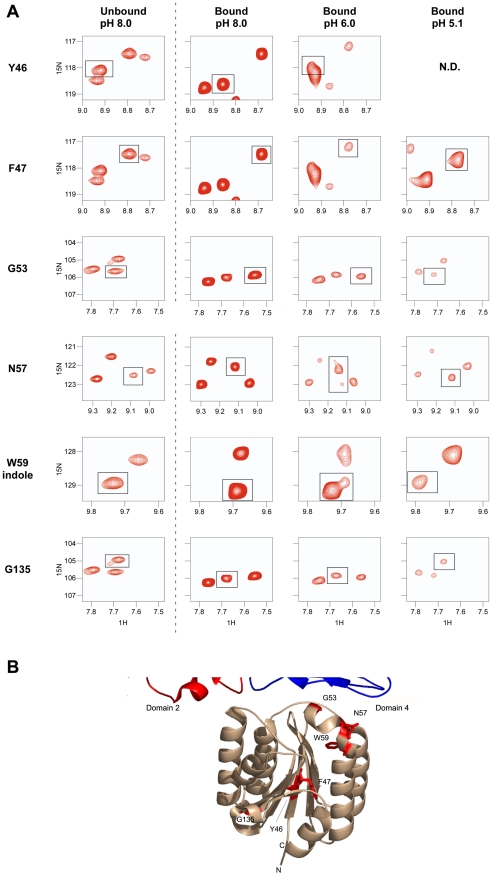
Chemical shift changes of the ANTXR2 spectra due to PA binding at several pH values. **A**) Chemical shift changes are shown for residues Y46, F47, G53, N57, the W59 indole, and G135, by comparing the unbound VWA domain at pH 8 (left panel) with the bound VWA-(PA_63_)_7_ complex at different pH values (right panels). N.D: Not Determined; Residue Y46 was not resolved at pH 5.1 **B**) Chemical shift perturbation of the ANTXR2 VWA domain upon PA_63_ heptamer binding. Chemical shift perturbations were seen for residues Y46, F47, G53, N57, the W59 indole, and G135 upon PA_63_ heptamer binding. These residues are modeled onto the crystal structure of the ANTXR2 VWA domain and highlighted in red.

The [^1^H,^15^N] TROSY-HSQC data also revealed chemical shift perturbations of certain receptor residues, including PA domain 4 contact-residues that were associated with (PA_63_)_7_ binding at pH 8.0 (Figure 6A). Small shift changes due to the isotope effect of being a highly deuterated protein were also taken into consideration as well as the pH effects [38]. Specifically, peaks associated with residues in and around helix 1 of the ANTXR2 VWA domain, that were involved in binding PA domain 4, significantly changed their position in the presence of PA_63._ These residues include G53, W59, and N57 (Figure 6B). A similar observation was made with residues Y46 and F47, which lie within the hydrophobic core of the ANTXR2 VWA domain, as well as with G135, which lies on the opposite face of the receptor VWA domain (Figure 6B). These latter effects are likely due to an allosteric or structural change in the receptor domain following PA binding. Strikingly, the peaks associated with all of these residues reverted back to their “unbound” configuration when the ANTXR2 VWA/PA_63_ complex was incubated at pH 5.1 (Figure 6A), even though the receptor remains bound to PA domain 4 under this condition. The only exception was residue Y46 which was not resolved at pH 5.1 but moved back towards its “unbound” configuration at pH 6, (Figure 6A).

## Discussion

**Figure 7:**
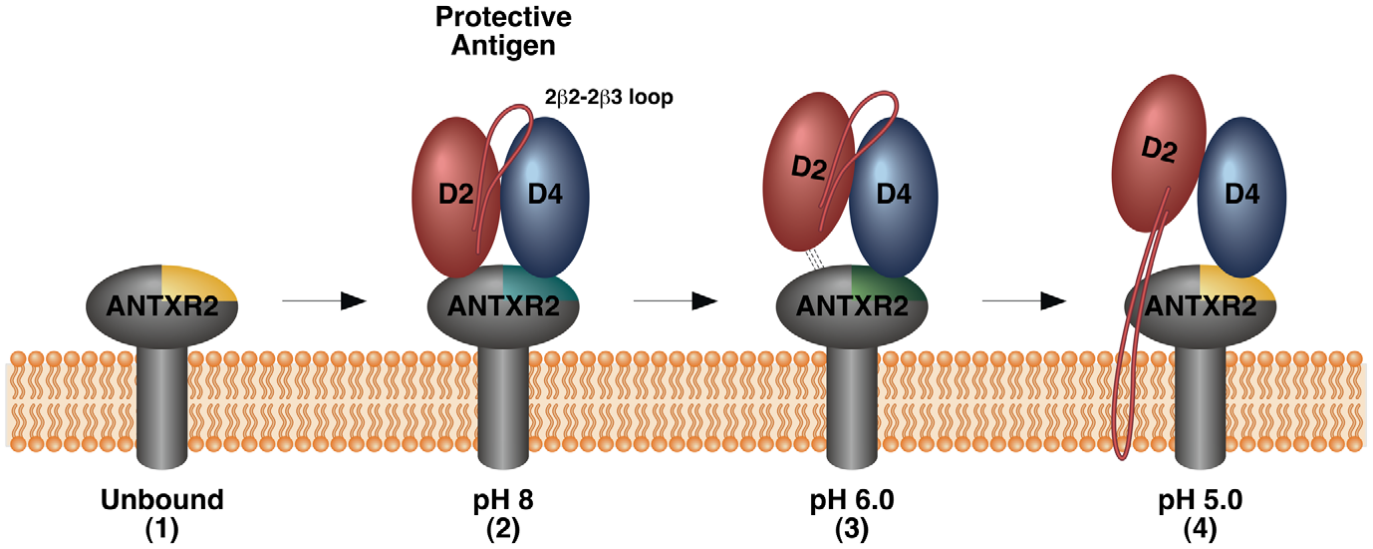
Model of changes in the PA-receptor contacts that accompany toxin prepore-to-pore conversion. For clarity, only domains 2 and 4 of a single PA monomer are shown with the receptor. 1. The unbound receptor with the PA Domain 4 binding site highlighted in yellow to indicate its “unbound” configuration. 2. The receptor binds to PA domains 2 and 4 forming a molecular clamp that blocks pore formation and inducing a conformational change in PA domain 4 contact residues (indicated with cyan shading). 3. At pH 6 which is similar to the conditions in an early endosomal compartment, the receptor contacts with PA domain 2 are weakened and PA domain 4 contact residues begin to revert back to their “unbound” configuration” (indicated with green shading). Additional allosteric effects are also detected at this pH value. 4. At ∼pH 5 which is similar to the conditions in a late endosomal compartment, PA domain 2 is no longer bound to receptor, presumably permitting movement of the 2β2-2β3 region of PA to mediate pore formation. The receptor remains bound to PA domain 4 after pore formation although certain PA domain 4 binding residues of the receptor revert back to their “unbound” configuration (indicated with yellow shading). The bound receptor may stabilize the structure and/or modify the function of the newly formed pore.

In this study we have used the TCS NMR approach to monitor how the ANTXR2-based receptor switch regulates anthrax toxin prepore-to-pore conversion. We showed that this is a robust method for identifying the receptor contacts with PA domains 2 and 4, in the prepore configuration at pH 8.0. We also obtained evidence at pH 6.0 for a new toxin-receptor intermediate in the pathway leading to pore formation, one in which the receptor remains bound to PA domain 4 but contacts with PA domain 2 have been significantly weakened. That intermediate would presumably exist within a mildly acidic early endosomal compartment during endocytic trafficking of toxin-receptor complexes [39]. Furthermore, we demonstrated that the ANTXR2 VWA-domain remains attached to PA domain 4 after triggering PA pore formation at pH 5.1, consistent with a more strongly acidic late endosomal pH [39]. Subtle structural changes, associated with reversion back to an unbound configuration, were also detected in residues located near the PA domain 4-binding site following pore conversion. This effect was also seen in the opposite face of the protein with residue G135, and with two hydrophobic residues within the core, Y46 and F47. It is known that chemical shifts of those nuclei that lie within close proximity of the binding partner can be substantially perturbed in the presence of that partner. However chemical shift perturbations (CSP) can also arise from allosteric effects as well as extended conformational changes that may occur in the target protein upon protein partner binding [40], [41]. These latter effects most likely account for the chemical shift perturbations seen with residues Y46, F47, and G135, which lie distal from the PA_63_ binding site of the receptor (Figure 6B). Taken together, these studies have led to a revised model of the changes in toxin-receptor contacts during pore formation (Figure 7) and support the idea that the bound receptor may influence the structural and/or functional properties of the toxin pore.

It is unlikely that the TCS effects that were observed could be attributed to non-specific aggregation of the PA_63_ heptamer-receptor complex at the different pH values tested since these effects were almost exclusively restricted to the toxin-binding face of the receptor. Indeed, inspection of 1D slices of the TCS experiments for selected crosspeaks did not indicate a broadening of lineshapes at several residues at pH 8 or pH 5, as would have be expected if there was aggregation (data not shown). Additionally, to further assess protein aggregation at pH 8 and pH 5, a wavelength scan (from 280–360 nm) was performed on the ANTXR2 VWA domain in complex with PA_63_ at the same 10∶1 ratio used for the TCS NMR experiments, since protein aggregation can be monitored at 340 nm [42], [43]. These studies revealed no substantial increase of absorbance at 340 nm between pH 8 and pH 5 (Supplementary Table S1), and visual inspection of the sample yielded no noticeable turbidity in the supernatant, under any of the conditions tested. Furthermore, there was no substantial difference in the absorbance at 280 nm seen with either the pH 8.0 or pH 5.0 samples before or after a 24 hour incubation at 37°C (Supplementary Table S2). Therefore, we concluded that the TCS NMR studies were not compromised by any non-specific aggregation of the PA63 heptamer-receptor complex at pH values ranging from pH 5–8.

Previous NMR studies had been interpreted as being consistent with receptor release from the newly formed toxin pore. In contrast to the current report, these studies included the detergent, octyl-glucoside and a higher salt concentration in the samples, and a lower temperature was used for the NMR experiments (293K versus 310K). In one of these studies, strongest methyl resonance of carbon-13 (SMRC) NMR analysis was employed, which analyzes the first dimension of a ^1^H- ^13^C heteronuclear single quantum coherence experiment (HSQC). That approach indicated that contacts between the PA pore and the ANTXR2 VWA-domain were lost at acidic pH [25]. Similar experiments were performed with a ^13^C-labeled 2 fluorohistidine labeled ANTXR2 VWA domain [25]. In both studies, a single peak was monitored upon PA binding, the ^1^H methyl resonance of ^13^C labeled ANTXR2 VWA domain, and was focused on the ^1^H methyl resonance (0.8 ppm) in the presence or absence of PA_63_. In the absence of PA_63_, a strong signal for the ANTXR2 VWA domain was seen as a sharp peak at 0.8 ppm, but in the presence of the toxin subunit, the peak signal was diminished because of line broadening, and there was substantial loss of peak height due to PA_63_ binding. These studies were conducted at both pH 8.0 and 5.0. Although the peak heights were increased at the lower pH value indicating PA_63_ dissociation, they did not return to the levels seen in the unbound state [25]. Therefore, we suggest that receptor dissociation might not have been complete when these studies were performed at pH 5.0. Consistent with the findings reporting the present report, another NMR study performed by the same group demonstrated that a recombinant fragment of PA (PA domain 4) remained bound to the ANTXR2 VWA-domain at pH 5.0 [27], although in that case it was not possible to relate these effects to the process of prepore-to-pore conversion.

Previous co-immunoprecipitation experiments led to conflicting conclusions about whether the receptor remains attached to the newly formed anthrax toxin pore complex. In one study, a PA-antiserum, that did not disrupt the PA_63_ prepore-receptor interaction, was used to demonstrate that both ANTXR1 and ANTXR2 co-precipitated with the PA_63_ prepore, but not with the PA_63_ pore [23]. In another study, an antiserum that recognized an epitope tag engineered into the cytoplasmic tail domains of both receptors co-precipitated both PA_63_ prepore and pore complexes [19], [26]. In light of the current report, it seems most likely that the latter study is correct and that these discrepant findings are probably due to the PA antiserum disrupting the weakened PA_63_-receptor interaction following pore formation. That effect would not be seen with antibodies binding to the cytoplasmic tails of the receptors. Therefore, these previous results obtained by co-immunoprecipitation of membrane-associated proteins are consistent with the conclusions of this report, i.e. both lines of evidence support receptor-association following prepore-to-pore conversion.

The bound receptor might influence the structural and/or functional integrity of the PA pore complex. The structure of the PA pore resembles the mushroom shaped structure of *S. aureus* α-hemolysin [44], [45]. However, the dimensions of these structures are drastically different. Crystallographic studies of the α-hemolysin pore revealed a mushroom structure with a 100 Å diameter cap and a stem region of 52 Å [44]. By contrast, electron micrograph studies of the PA pore stabilized with GroEL revealed a 125 Å diameter cap and a stem region that was almost as long (100 Å) [45]. Santelli and colleagues hypothesized that the receptor might occupy the predicted 75 Å gap between the pore cap structure and the membrane, thereby stabilizing the pore [16]. The results of the present study, which demonstrates that receptor remains bound to PA domain 4 after pore formation is triggered, provide direct support for a possible pore-stabilizing role for the receptor.

A pore-stabilizing role for the receptor is also consistent with results from a previous voltage patch clamp study of ion conductance by the PA pore in whole cells versus artificial membranes. That study indicated that the receptor might influence pore structure since it was associated with altered voltage-dependent inactivation properties of the pore and with altered sensitivity to inhibition by the small molecule inhibitor, TBA [28]. Also consistent with such a role, it has been reported that disulfide-bond formation in the extracellular immunoglobulin-like region of ANTXR2, which lies between the membrane and the VWA-domain of the receptor, can influence anthrax toxin pore function [46]. Future studies will aim to uncover how the receptor-PA domain 4 contacts influence the structure and or function of the anthrax toxin pore.

## Materials and Methods

### Protein expression and purification

The VWA-domain of ANTXR2 (residues Ser38 to Cys218) was produced from a pGEX-4T-1 vector (Amersham Pharmacia) and was expressed as a GST fusion protein [47] in *Escherichia coli* C43 (DE3) cells (OverExpress). The RIL plasmid of BL21-CodonPlus-RIL cells (Stratagene) was also co-expressed in the C43 (DE3) cells due to rare codons within the VWA-domain protein-encoding region. Isotopically enriched ^15^N, ^15^N/^13^C/^2^H, ^15^N/^13^C, and ^15^N/^2^H ANTXR2 VWA-domain samples were prepared for NMR studies from 4 liters of *E. coli* culture grown in standard M9 minimal media with ^15^NH_4_Cl at 0.1% (wt/vol), with and without ^13^C_6_-glucose or ^2^H/^13^C-glucose (0.4% (wt/vol). Unlabeled samples of the ANTXR2 VWA-domain were produced in standard Terrific Broth. The cell cultures were grown with carbenicillin (50 ug/ml), chloramphenicol (34 ug/ml), and spectinomycin (50 ug/ml) for plasmid selection.

For the transfer cross saturation (TCS) experiments, the ANTXR2 VWA-domain was produced in 100% D_2_O based M9 minimal media supplemented with ^15^NH_4_Cl (0.1% wt/vol), ^2^H/^13^C_6_-glucose (0.4% wt/vol) and MEM Vitamin B solution (Sigma). Growth of the C43 (DE3) cells in 100% D_2_O required acclimating the cells in 5 mls of standard M9 minimal media and slowly acclimating the cells to a 20% increase in D_2_O levels every 12–24 hours until growth was sustained in 100% D_2_O-containing medium. A 5 ml sample of cells grown in 100% M9 media was then used to inoculate 1L of 100% D_2_O M9 media, which was then used for standard isotopic labeling procedures. Once the cell populations had reached an OD_600_ of 0.75, ANTXR2 VWA-domain expression was induced with 0.5 mM isopropyl β-d-thiogalactopyranoside (IPTG) for 6–8 hours at 37°C. The bacterial cells were then harvested by centrifugation at 8000× g in a JA-10 rotor and resuspended into 50–75 ml of lysis buffer (50 mM Tris pH 7.5; 150 mM NaCl; 1 mg/ml lysozyme, 100 units DNAse). The cells were then lysed by three cycles of sonication (0.5 sec pulses/20 seconds per cycle using a 550 Sonic Dismembrator (Fisher Scientific)) and protease inhibitor cocktail II tablets (Roche) were added to the lysate. The lysate was cleared by centrifugation at 12,000× g in a JA-20 rotor for 1 hour at 4°C and the supernatant was filtered with a 45 µm filter (vacuum filtration device (Nalgene)). The supernatant was circulated over a 5 ml GSTrap HiTrap FF column (Amersham Pharmacia) using a peristaltic pump (LKB Pump P1, Amersham Pharmacia). The resin was then washed with Buffer A (50 mM Tris HCl pH 8.0; 150 mM NaCl) and incubated with 5 mls of thrombin cleavage buffer (50 mM Tris HCl pH 8.0; 150 mM; 5 mM CaCl_2_; 500 units thrombin (Sigma)) for 12–16 hours overnight at room temperature. The labeled protein samples were eluted with Buffer A and cleared of thrombin using a HiTrap Benzamidine FF column (Amersham Pharmacia). The protein was further concentrated using a filtered centrifugal device (Vivaspin 15R, Sartorius).

A lack of several backbone amide resonances in the [^15^N,^1^H] TROSY-HSQC of the ANTXR2 VWA-domain was observed and attributed to slow back exchange of the amides from deuterons to protons, when the protein expressing *E. coli* were grown in a D_2_O based media. Because this phenomenon resulted in the loss of several probes, the deuterium-labeled ANTXR2 VWA-domain had to be unfolded to protonate the deuterated residues that were buried within the core of the folded protein. ANTXR2 VWA-domain was unfolded at a concentration of 1 mg/ml and protein unfolding was performed for 1 hour at 4°C in unfolding buffer (3M guanidine HCl; 50 mM Tris-HCl pH 8.0; 150 mM NaCl). It was then added drop-wise with stirring into refolding buffer (50 mM Tris-HCl; 2 mM MgCl_2_; 150 mM NaCl; 10% vol/vol glycerol) at 4°C, and kept under agitation for one hour. The refolded protein sample was then dialyzed against NMR buffer (50 mM Tris-HCl pH 8.0; 150 mM NaCl) and concentrated using a filtered centrifugal device (Vivaspin 15R, Sartorius). The integrity of the refolded protein was demonstrated by a [^15^N,^1^H] TROSY-HSQC which was comparable to a control spectrum of a non-denatured ^1^H-^15^N ANTXR2 VWA-domain, and through an in vitro toxin neutralization assay as described elsewhere [48].

PA_83_ was expressed from a pET22b^+^ vector (Novagen) [49] in Rosetta 2 cells (Novagen) due to rare codon usage and grown at 37°C in Luria Broth containing carbenicillin (50 ug/ml) and chloramphenicol (34 ug/ml). Cells were grown to an OD_600_ of 1.0, and PA_83_ expression was induced by addition of 0.5 mM IPTG for 6 h at 25°C. Periplasmic proteins were obtained by osmotic shock by first resuspending pelleted cells in 1L of Buffer B (20% sucrose; 5 mM EDTA; 50 mM Tris-HCl (pH 8.0)) with stirring at room temperature for ten minutes. The cells were then harvested at 8000× g for 15 minutes at 4°C in a JA-10 rotor and the pellet was resuspended with stirring in a cold 5 mM MgSO_4_ solution at 4°C for 15 minutes. This sample was centrifuged again with the same harvesting conditions, protease inhibitor tablets (Roche) were added and the resulting supernatant containing the desired protein was brought up to 50 mM Tris HCl at pH 8.0 with a 1M stock solution of Tris HCl pH 8.0. The supernatant was then circulated over an anion-exchange HiTrap QFF column (Amersham Pharmacia) and purified with a gradient of 0M to 1M NaCl in buffer A (50 mM Tris-HCl; pH 8.0) using an AKTA-FPLC system (Amersham Pharmacia). Column fractions containing PA_83_ were then concentrated and applied to a Hi Load Superdex 26/60 gel filtration column (Amersham Pharmacia) and eluted using gel filtration buffer (50 mM Tris-HCl; 150 mM NaCl; pH 8.0). PA_83_ was purified to 90% homogeneity, as judged by a Coomassie stained SDS-PAGE gel and concentrated using centrifugal filter devices (Vivaspin 15R, Sartorius). This protocol was modified from Miller *et al* 1999 in order to produce a large scale prep for NMR studies [49].

To generate PA_63_ by trypsin cleavage [49], the purified PA_83_ sample was concentrated down to 1.5 ml (final concentration 5 mg/ml) for treatment with trypsin-conjugated magnetic beads. Prior to that incubation, 1 ml of the magnetic beads slurry (Mag-Trypsin, Clontech) was washed in gel filtration buffer and then separated from the wash using a microfuge magnetic stand (Promega). The washed beads were then mixed with the purified PA_83_ for 45 minutes at room temperature with constant agitation (Nutator). The trypsin beads were then removed using the magnetic stand and the generated PA_63_ heptamer was purified by gel filtration using a Hi Load Superdex 26/60 gel filtration column (Amersham Pharmacia) and samples were eluted with gel filtration buffer (50 mM Tris-HCl; 150 mM NaCl; pH 8.0). Fractions containing the heptamer were then concentrated by a filtered centrifugal device (Vivaspin 15R, Sartorius). The composition of the PA_63_ heptamer was confirmed by static light scattering/refractive index measurements coupled with size exclusion chromatography (data not shown).

### Circular Dichroism spectroscopy of ANTXR2

The ANTXR2 VWA-domain was purified as described, and the samples were concentrated to 10 mg/mL and stored at 4°C. The concentrated samples were diluted into either Buffer A (50 mM Tris-HCl, pH 8.0; 5 mM DTT, 150 mM NaCl, 2.5 mM MgCl_2_) or into Buffer B (50 mM sodium phosphate buffer, pH 5.0 or 6.0; 150 mM NaCl; 2.5 mM MgCl_2_) to a final concentration of 25 µM, Supplementary Figures S1 and S2, respectively. The solution was then placed into a 0.1-cm path-length quartz cell (Hellma, Forest Hills, NY). Spectra were acquired using a BioLogic MOS-450 (Molecular Kinetics, Pullman, WA). All measurements were done at 25°C. Spectra were recorded at a wavelength range of 190–260 nm. Three independent experiments were performed with each sample. Raw data were manipulated by smoothing and subtraction of buffer spectra, according to the manufacturer's instructions.

### Assessment of in vitro aggregation

300 µM of ANTXR2 VWA domain was incubated at 37°C for 48 hours in buffers ranging from pH 8, 7, 6, and 5. Buffer A (50 mM Tris-HCl, 150 mM NaCl, 2.5 mM MgCl_2_) was used for the pH 8 and pH 7 samples. Buffer B (50 mM phosphate buffer; 150 mM NaCl, 2.5 mM MgCl_2_) was used for and the pH 6 and pH 5.1 samples. The samples were subjected to a wavelength scan using a Beckman DU 530 Life Science UV/VIS spectrophotometer. The apparent optical density, which is proportional to turbidity, was then analyzed at 340 nm.

### Assignment of the ANTXR2 VWA-domain

All NMR experiments were recorded at 310K on a Bruker 700-MHz spectrometer equipped with four radiofrequency channels and a triple-resonance cryoprobe with a shielded z-gradient coil. Measurements were performed on either ^15^N, ^15^N/^13^C, or ^15^N/^13^C/^2^H, 350 µM labeled ANTXR2 VWA-domain in NMR buffer (50 mM Tris-HCl (pH 8.0); 150 mM NaCl; 0.01% NaN_3_; 10/90 D_2_O/H_2_O), if not stated otherwise. Assignments for the backbone resonances were obtained using data from the following experiments: [^1^H,^15^N] TROSY-HSQC, 3D TROSY-HNCO, 3D TROSY-HN(CA)CO, 3D TROSY-HNCACB, 3D TROSY-HNCA, and a 3D ^15^N-edited NOESY-HSQC. NMR data were processed using NMRPipe and analyzed using the Sparky and CARA software packages [34], [35].

### Transferred cross saturation experiments

Transferred cross-saturation experiments were performed with deuterated, ^15^N-labeled ANTXR2 VWA-domain in a buffer containing 85-90% D_2_O. The final NMR sample contained 350 µM VWA-domain and 35 µM PA_63_ (ratio 10∶1) in NMR buffer (50 mM deuterated Tris-HCl pH 8.0 buffer; 5 mM DTT; 150 mM NaCl; 2.5 mM MgCl_2_; 85% D_2_O/H_2_O) or low pH NMR buffer (50 mM sodium phosphate buffer pH 6.0 or 5.1; 150 mM NaCl; 2.5 mM MgCl2; 85% D_2_O) Experiments were performed at 310K. Selective saturation of the protein was achieved by applying a train of Gaussian shaped pulses prior to the [^1^H-^15^N] TROSY-HSQC experiment with the saturation frequency set to 0.8 ppm [32], [50]. The experiment was performed in an interleaved manner with a phase sensitive Echo/Antiecho gradient selection. Experiments were performed similarly with 120 scans, 0.5 sec saturation durations, and a relaxation delay of 2.0 s. The experiments were performed with 2048× 256 complex points in the ^1^H and ^15^N dimensions with spectral widths of 10000 and 2270 Hz, respectively. The spectra were transformed to 2048× 256 complex points using zero-filling.

## Supporting Information

Figure S1 Far-UV CD spectrum of the refolded ANTXR2 VWA-domain in **A)** 50 mM Tris-HCl buffer, pH 8.0, **B)** 50 mM sodium phosphate buffer, pH 6.0, and **C)** 50 mM sodium phosphate buffer, pH 5.0
(TIF)

Figure S2 The refolded, double-labeled ANTXR2 VWA-domain acts as an efficient receptor decoy that protects RAW264.7 cells against intoxication by anthrax lethal toxin.
(TIF)

Figure S3 Selected cross-sections of the HNCACB showing connectivity between several backbone residues of the ANTXR2 VWA-domain. Additional spectra were also obtained for backbone assignment.
(TIF)

Figure S4 PA_63_ heptamer forms a SDS-resistant species that is consistent with the pore in the presence of ANTXR2 VWA-domain at an acidic pH of <5.2. PA_63_ at 15 µM was incubated with 150** µ**M ANTXR2 VWA domain overnight at 37°C at the various pH values shown. The samples were then subjected to SDS-PAGE. As shown, in the presence of ANTXR2 VWA domain, the SDS resistant PA63 heptameric pore was formed only at pH<5.2.
(TIF)

Table S1 Turbidity of the ANTXR2:PA complex at a 10∶1 ratio. A sample containing 200 mM ANTXR2 and 20 mM (PA_63_)_7_ was incubated at 37°C and either pH 8.0 or pH 5.1 for 24 hours and the absorbance values at 340 nm were measured.
(TIF)

Table S2 Protein absorbance of ANTXR2:PA after 24 hours incubation at 37°C. A 250 mL sample containing 200 mM ANTXR2 and 20 mM (PA_63_)_7_ was incubated at 37°C and either pH 8.0 or pH 5.1 for 24 hours. The samples were then centrifuged using a table top centrifuge (Eppendorf Centrifuge 5424) at 13,000 rpm for 1 minute and the protein concentrations in the supernatants were measured at A_280_ nm. SDS-PAGE analysis was used to confirm that the PA heptamer remained in solution (data not shown).
(TIF)
