# Supplementary figures and images for: A Receptor-based Switch that Regulates Anthrax Toxin Pore Formation
Source: PLoS Pathog. 2011 Dec 8;7(12):e1002354. doi: 10.1371/journal.ppat.1002354 (PMC3234216; doi:10.1371/journal.ppat.1002354)

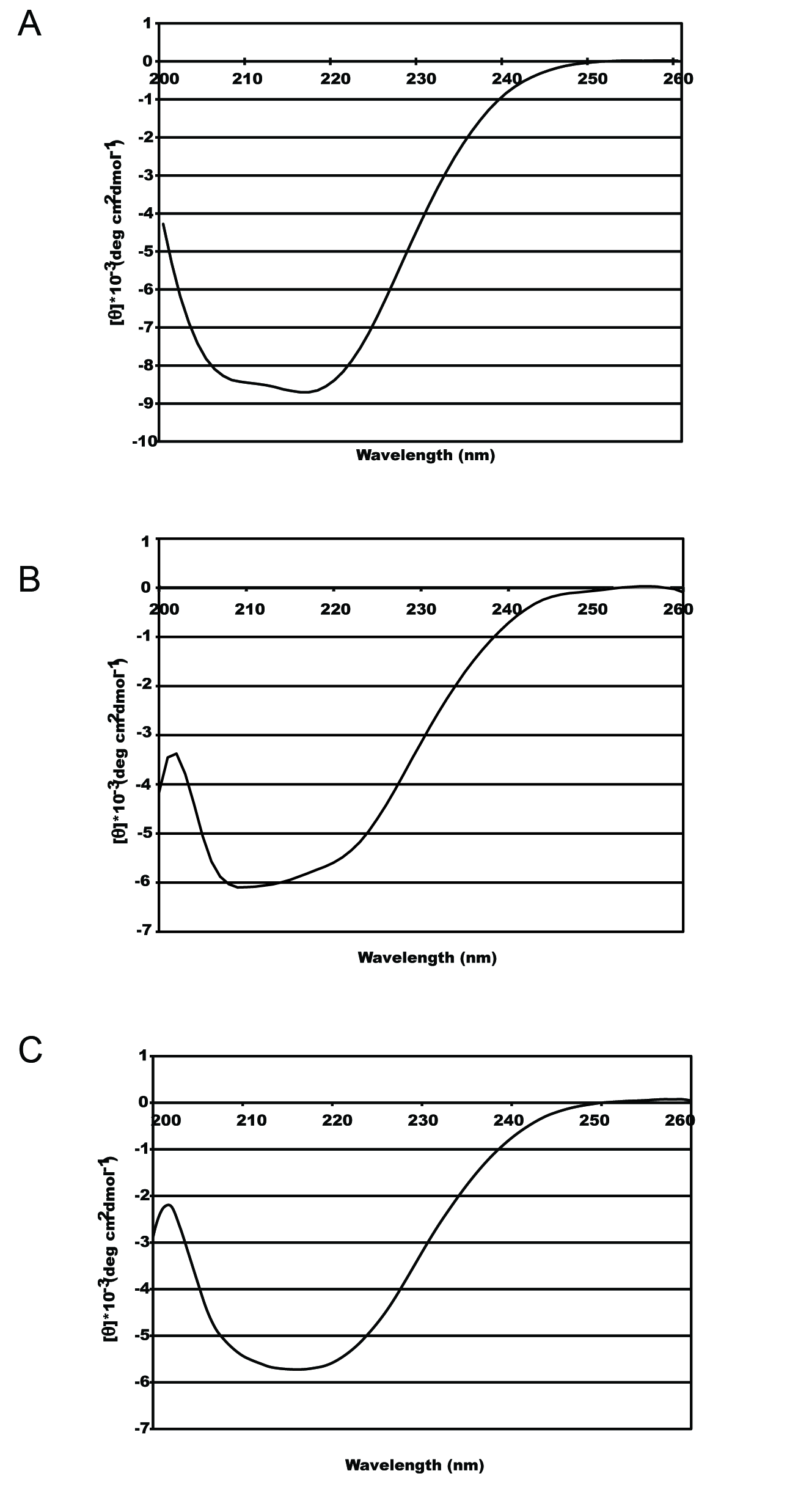

Supplement: Figure S1 — Far-UV CD spectrum of the refolded ANTXR2 VWA-domain in A) 50 mM Tris-HCl buffer, pH 8.0, B) 50 mM sodium phosphate buffer, pH 6.0, and C) 50 mM sodium phosphate buffer, pH 5.0 (TIF) [file ppat.1002354.s001.tif]

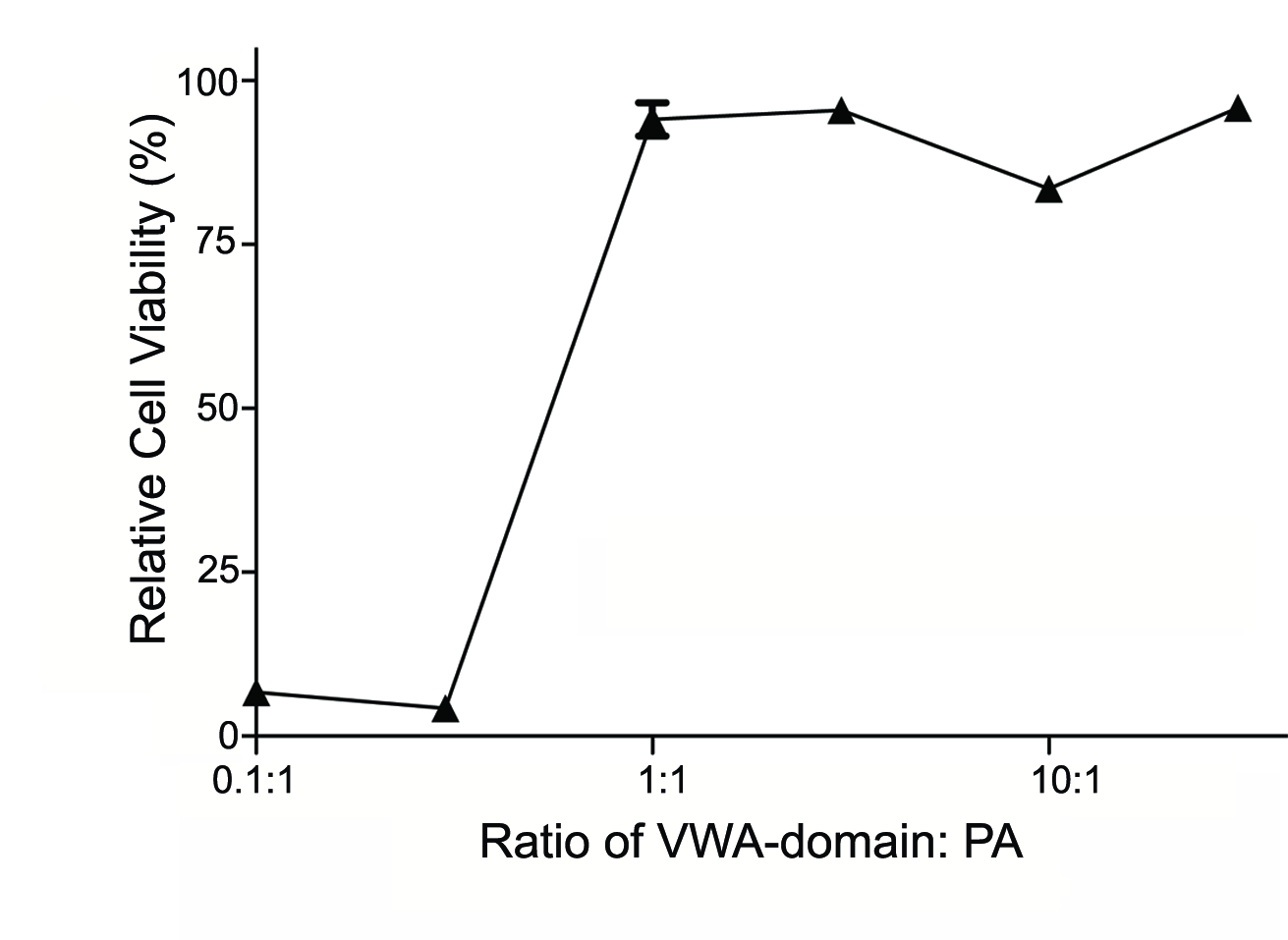

Supplement: Figure S2 — The refolded, double-labeled ANTXR2 VWA-domain acts as an efficient receptor decoy that protects RAW264.7 cells against intoxication by anthrax lethal toxin. (TIF) [file ppat.1002354.s002.tif]

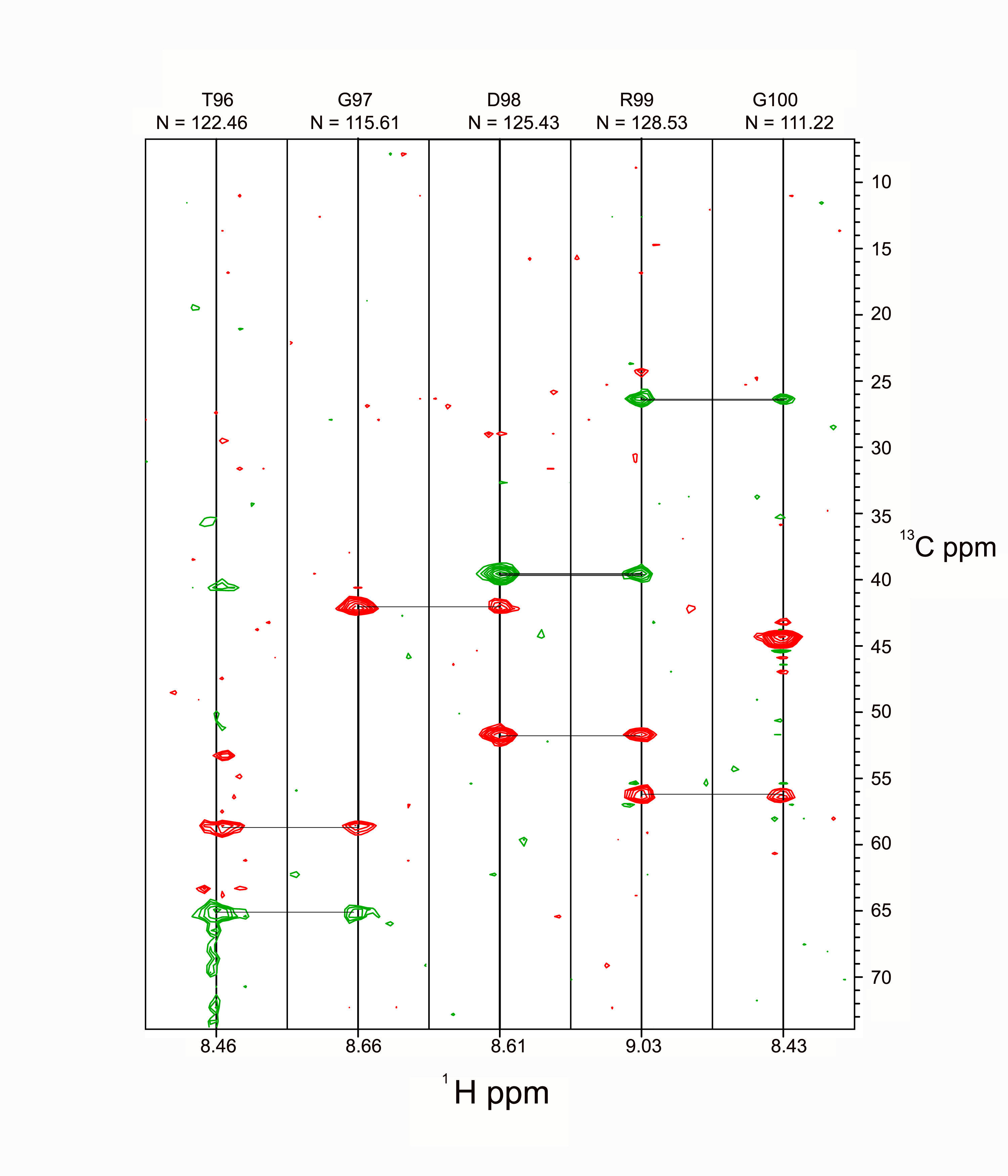

Supplement: Figure S3 — Selected cross-sections of the HNCACB showing connectivity between several backbone residues of the ANTXR2 VWA-domain. Additional spectra were also obtained for backbone assignment. (TIF) [file ppat.1002354.s003.tif]

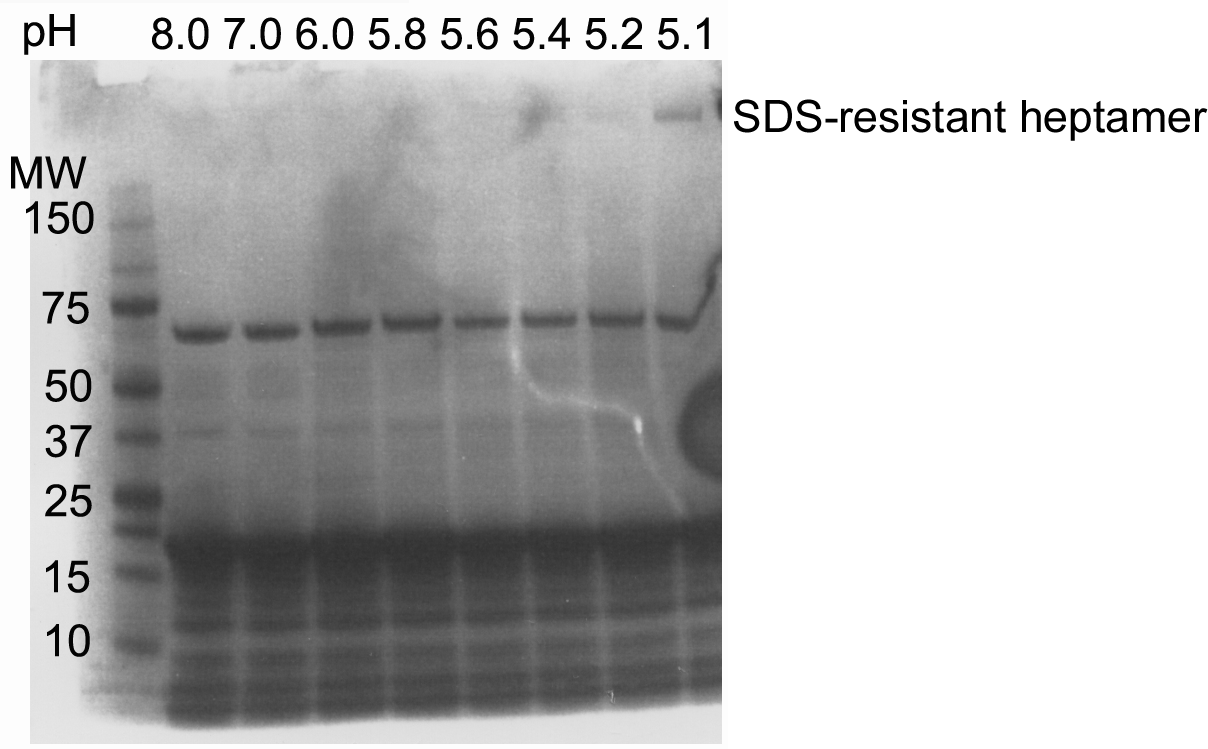

Supplement: Figure S4 — PA63 heptamer forms a SDS-resistant species that is consistent with the pore in the presence of ANTXR2 VWA-domain at an acidic pH of <5.2. PA63 at 15 µM was incubated with 150 µM ANTXR2 VWA domain overnight at 37°C at the various pH values shown. The samples were then subjected to SDS-PAGE. As shown, in the presence of ANTXR2 VWA domain, the SDS resistant PA63 heptameric pore was formed only at pH<5.2. (TIF) [file ppat.1002354.s004.tif]

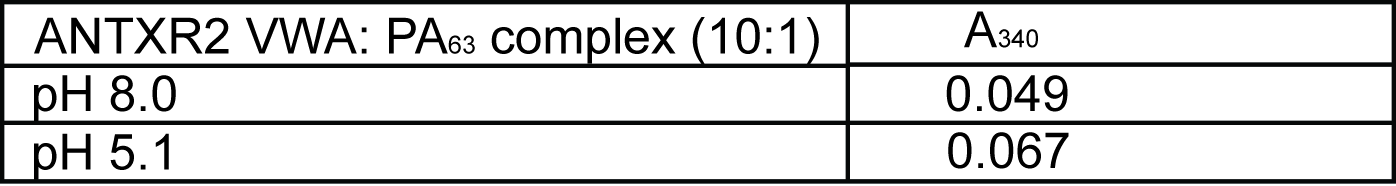

Supplement: Table S1 — Turbidity of the ANTXR2:PA complex at a 10∶1 ratio. A sample containing 200 mM ANTXR2 and 20 mM (PA63)7 was incubated at 37°C and either pH 8.0 or pH 5.1 for 24 hours and the absorbance values at 340 nm were measured. (TIF) [file ppat.1002354.s005.tif]

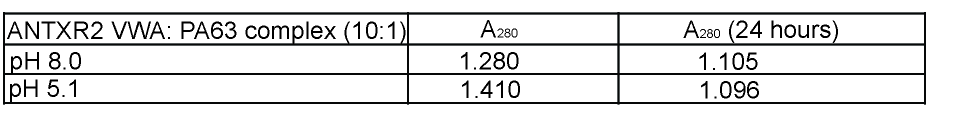

Supplement: Table S2 — Protein absorbance of ANTXR2:PA after 24 hours incubation at 37°C. A 250 mL sample containing 200 mM ANTXR2 and 20 mM (PA63)7 was incubated at 37°C and either pH 8.0 or pH 5.1 for 24 hours. The samples were then centrifuged using a table top centrifuge (Eppendorf Centrifuge 5424) at 13,000 rpm for 1 minute and the protein concentrations in the supernatants were measured at A280 nm. SDS-PAGE analysis was used to confirm that the PA heptamer remained in solution (data not shown). (TIF) [file ppat.1002354.s006.tif]
